# Nucleotide transmitters ATP and ADP mediate intercellular calcium wave communication via P2Y_12/13_ receptors among BV-2 microglia

**DOI:** 10.1371/journal.pone.0183114

**Published:** 2017-08-11

**Authors:** Pengchong Jiang, Fulin Xing, Bu Guo, Jianyu Yang, Zheming Li, Wei Wei, Fen Hu, Imshik Lee, Xinzheng Zhang, Leiting Pan, Jingjun Xu

**Affiliations:** 1 The Key Laboratory of Weak-Light Nonlinear Photonics, Ministry of Education, TEDA Institute of Applied Physics and School of Physics, Nankai University, Tianjin, China; 2 Collaborative Innovation Center of Extreme Optics, Shanxi University, Taiyuan, Shanxi, China; 3 The 2011 Project Collaborative Innovation Center for Biological Therapy, Nankai University, Tianjin, China; Scuola Superiore Sant'Anna, ITALY

## Abstract

Nerve injury is accompanied by a liberation of diverse nucleotides, some of which act as ‘find/eat-me’ signals in mediating neuron-glial interplay. Intercellular Ca^2+^ wave (ICW) communication is the main approach by which glial cells interact and coordinate with each other to execute immune defense. However, the detailed mechanisms on how these nucleotides participate in ICW communication remain largely unclear. In the present work, we employed a mechanical stimulus to an individual BV-2 microglia to simulate localized injury. Remarkable ICW propagation was observed no matter whether calcium was in the environment or not. Apyrase (ATP/ADP-hydrolyzing enzyme), suramin (broad-spectrum P2 receptor antagonist), 2-APB (IP_3_ receptor blocker) and thapsigargin (endoplasmic reticulum calcium pump inhibitor) potently inhibited these ICWs, respectively, indicating the dependence of nucleotide signals and P2Y receptors. Then, we detected the involvement of five naturally occurring nucleotides (ATP, ADP, UTP, UDP and UDP-glucose) by desensitizing receptors. Results showed that desensitization with ATP and ADP could block ICW propagation in a dose-dependent manner, whereas other nucleotides had little effect. Meanwhile, the expression of P2Y receptors in BV-2 microglia was identified and their contributions were analyzed, from which we suggested P2Y_12/13_ receptors activation mostly contributed to ICWs. Besides, we estimated that extracellular ATP and ADP concentration sensed by BV-2 microglia was about 0.3 μM during ICWs by analyzing calcium dynamic characteristics. Taken together, these results demonstrated that the nucleotides ATP and ADP were predominant signal transmitters in mechanical stimulation-induced ICW communication through acting on P2Y_12/13_ receptors in BV-2 microglia.

## Introduction

The intracellular roles of nucleotides (ATP, ADP, UTP, etc.) have long been recognized as energy currency or building blocks for DNA and RNA. However, in the extracellular compartment, mounting evidences reveal that they function as key signal molecules to participate in various processes including inflammation, infection and tissue damage through activation of membrane P2 nucleotide receptors [1, 2]. For instance, during nerve injury, amount of ATP, ADP and UTP liberated from apoptotic neurons and acted as ‘find-me’ signal via activating P2Y receptors, resulting in oriented migration of microglia [3, 4]. The ‘eat-me’ signal such as UDP will promote phagocytic clearance of dead cells and tissue debris by microglia through activating P2Y_6_ receptor [5, 6]. Moreover, extracellular nucleotide signaling by P1 and P2 receptors was found to regulate numerous inflammatory responses, which contributed to maintain the pro/anti-inflammatory balance of the central nervous system (CNS) [1, 7].

In the CNS, microglia acted as the first and main immune defense against infectious and pathological events [8]. They could promptly survey changes of the surroundings and sense extracellular messengers via cell surface receptors [9], subsequently congregate together rapidly to build a protective barrier between normal and damaged tissues [10]. Intercellular Ca^2+^ wave (ICW) communication was the principal approach by which microglia [11, 12], astrocytes [13], and neurons [14] coordinate and synchronize with each other to execute immune defense and maintain homeostasis of CNS. ICWs could prefigure and guide the migration of microglia in response to neuronal damage [15].

Many reports showed that extracellular nucleotide ATP played as an important signal transmitter in ICWs [16–18]. However, it remains unclear whether and how other types of extracellular nucleotides contribute to ICW communication in microglia. In the present study, we evoked ICWs in an immortalized murine microglial cell line (BV-2) by localized micro-stimulation on single cell using glass microelectrodes. We investigated the involvement of potential intercellular messengers and corresponding receptors. Results provided compelling evidences that both ATP and ADP played pivotal roles in ICW communication via P2Y_12/13_ receptors in BV-2 microglia, rather than other types of nucleotides.

## Materials and methods

### Cell culture

BV-2 cell is an immortalized mouse microglial cell line that exhibits the morphological and functional characteristics of microglia [19]. The BV-2 cells, a generous gift from Dr. Linhua Jiang (Institute of Membrane and System Biology, Faculty of Biological Sciences, University of Leeds, Leeds, U.K.), were routinely cultured in Dulbecco’s modified Eagle’s medium (DMEM, Gibco, USA) supplemented with 10% (v/v) fetal bovine serum (FBS, Biological Industries, USA), 100 U/mL penicillin and 100 μg/mL streptomycin (Gibco, USA) at 37°C under 5% CO_2_. Before test, cells were isolated with 0.25% trypsin + 0.04% EDTA (Gibco, USA) and plated on glass coverslips at a density of 2×10^4^ cells/cm^2^ to be adherent for 18 h.

### Single-cell level mechanical stimulation

Local mechanical stimulus was performed using glass microelectrodes (1 μm tip diameter), drawn by flaming/brown micropipette puller (Model P-97-6368, Sutter Instrument Co., USA). The glass microelectrodes were mounted on a Three-axis Hanging Joystick Oil Hydraulic Micromanipulator (MMO-202ND, Narishige, Japan), which was fixed on the Axio observer D1 inverted fluorescent microscope (Carl Zeiss, Germany). During fluorescence image acquisition, the tip of the microelectrode was controlled to provoke an acute, short-lasting mechanical stimulation on target cells.

### Ca^2+^ imaging

Measurements of cytosolic calcium concentration ([Ca^2+^]_c_) in microglia was performed as described previously [11]. Briefly, BV-2 microglial cells were incubated with 3 μM calcium-sensitive Fluo-4 AM (Invitrogen, USA) for 40 min at 37°C in Hanks balanced salt solution (HBSS, 140 mM NaCl, 5 mM KCl, 2 mM CaCl_2_, 1 mM MgCl_2_·6H_2_O, 10 mM glucose, 10 mM HEPES, pH 7.4) in the presence of 10% pluronic F-127 (Biotium, USA), subsequently washed for 10 min with HBSS for de-esterification. Ca^2+^ dynamics was observed by the Axio observer D1 inverted fluorescent microscope. Fluo-4 was excited by a mercury with a 485/20 nm excitation filter, and fluorescence was collected by a fluar 40×/1.30 oil objective with 540/50 nm emission filter. Images were acquired by an electron multiplying charge coupled device (EMCCD) (DU-897D-CS0-BV, Andor, U.K.), which was controlled by MetaMorph software (Universal Imaging Corp., USA). Each fluorescence image was acquired for 50 ms with a 1 s interval between frames. The obtained data were quantitatively analyzed for changes of fluorescence intensities within the region of interest (ROI). The [Ca^2+^]_c_ level over time was presented as relative fluorescence intensity (F/F_0_, intensity after stimulation/basal intensity before stimulation).

### RT-PCR

Total RNA from BV-2 microglial cells was isolated using RNAprep pure Cell/Bacteria Kit (Tiangen, Beijing). Then, the RNA (1 μg) was subjected to reverse transcription (RT) using a RT system (Promega, USA) in a total volume of 20 μl that contained MgCl_2_ (25 mM, 4 μl), reverse transcription 10× buffer (2 μl), dNTP mixture (10 mM, 2 μl), RNase inhibitor (0.6 μl), AMV reverse transcriptase (1 μl) and Oligo(dT) primer (1 μl). The reaction mixtures were incubated at 45°C for 30 min, 99°C for 5 min to inactivate the enzyme, and then chilled on ice for 5 min. Subsequently, the product of RT reaction (1 μl) was amplified using a GoTaq Green Master Mix (Promega, USA) in a total volume of 50 μl PCR buffer containing Green Master Mix, 2× (25 μl), sense primer (0.5 μl) and antisense primer (0.5 μl). The reaction mixtures were preheated to 95°C for 2 min followed by 40 thermal cycles in a PCR machine (MJMiniTM, BIO-RAD, USA). For each cycle, denaturation was at 95°C for 30 s, annealing at 63.5°C for 30 s, and extension at 72°C for 1 min. Sequences of gene-specific primers (S1 Table) were designed using the Primer 5 (Premier Biosoft, USA) and evaluated with Oligo 7 (Molecular Biology Insights, USA).

### Western blot

BV-2 microglia were seeded in a 6-well plate and lysed in 100 μL RIPA solution (Beyotime, China). The total protein concentration was determined by BCA assay. Total protein lysates (~80 μl) were denatured at 95°C for 10 min. Proteins (20 μg/lane) were subjected to 8% SDS-PAGE by 80 V at room temperature for 2 h. The resolved proteins were transferred onto nitrocellulose membranes by 80 V for 1.5 h. Upon blocking non-specific binding sites with 3% BSA for 1 h at 4°C, membranes were incubated using the following primary antibodies overnight at 4°C: rabbit anti-P2Y_12_ (1:500; 75 kDa; Alomone, Isreal) and mouse anti-β-actin (1:1000; 42 kDa; Beyotime, China). After washing, membranes were incubated with horseradish peroxidase-labeled goat anti-rabbit (for P2Y_12_; 1:1000; Alomone, Isreal) and goat anti-mouse (for β-actin; 1:1000; Beyotime, China) secondary antibodies for 1h. Finally, bands were visualized with the enhanced chemiluminescence (ECL; Tanon, China) and the Tanon 5200 MultiImage System (Tanon, China).

### Immunofluorescence

BV-2 microglial cells were prepared as described above and seeded on a glass cover slip in a 6-well plate (Corning, USA) for 24 h. For living cell immunostaining, cells were incubated with anti-P2Y_12_ (extracellular) rabbit antibody (1:500; Alomone, Israel) resolved in DMEM at 37°C for 1h, followed by washing with HBSS for three times. Then, cells were stained with Alexa-488 goat anti-rabbit IgG (1:500; Abcam, UK) at 37°C for 1h. After washing with HBSS for three times, images were obtained by 535 nm emission filter based on an Olympus IX51 inverted fluorescence microscope with 40× oil objective.

### Statistical analysis

A cell was defined as responsive to mechanical stimulation if it showed a calcium transient with a peak magnitude not less than 1.1 times of baseline. The response rate was defined as the number of responsive neighboring cells (except the stimulated cell) divided by the total number of neighboring cells in each group. All data were analyzed using the IBM software SPSS (version 22). For multiple comparisons, the data were analyzed using unpaired Student’s t-test and analysis of variance (ANOVA) followed by the Scheffé *post hoc* test. *P* < 0.05 was considered statistically significant.

## Results

### Mechanical stimulation on a single BV-2 microglia initiates intercellular Ca^2+^ wave propagations

**Fig 1:**
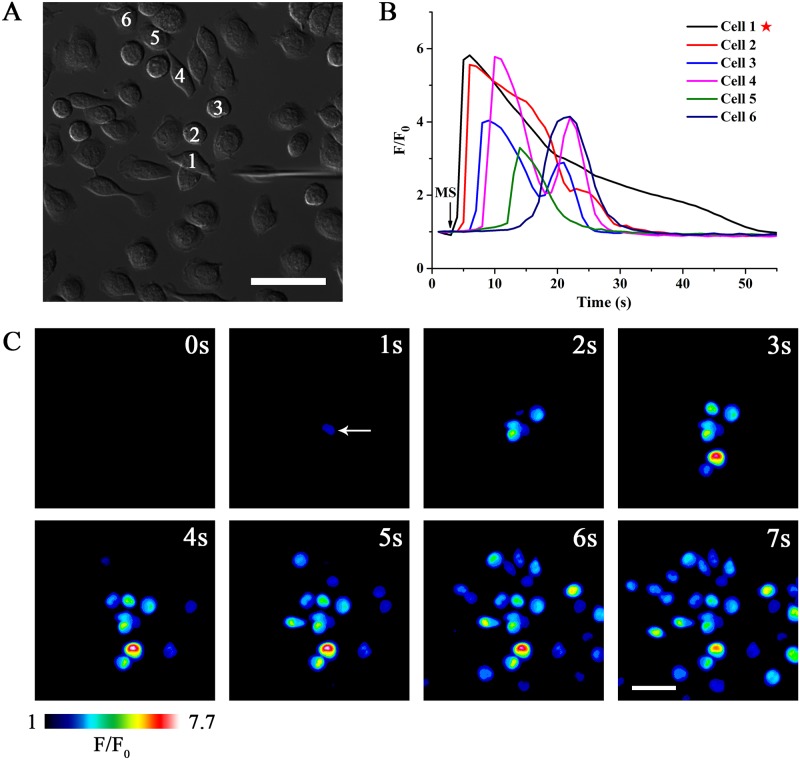
Mechanical stimulation on an individual cell induces intercellular calcium wave (ICW) propagations among separated BV-2 microglia. MS indicates the mechanical stimulation event. Pentagram indicates the stimulated cell. **(A)** Representative image of BV-2 microglia and glass microelectrode visualized by differential interference contrast (DIC) microscope. **(B)** The Ca^2+^ traces of the stimulated cell (Cell 1) and five neighboring cells (Cell 2–6). **(C)** A sequence of pseudocolor images that show mechanical stimulation elicited-propagation of ICWs among isolated BV-2 microglia. The white arrow indicates the application of mechanical stimulation on the selected microglia. Relative changes in [Ca^2+^]_c_ are depicted on a pseudocolor scale with white representing the highest and black the lowest values. Scale Bar = 50 μm.

**Fig 2:**
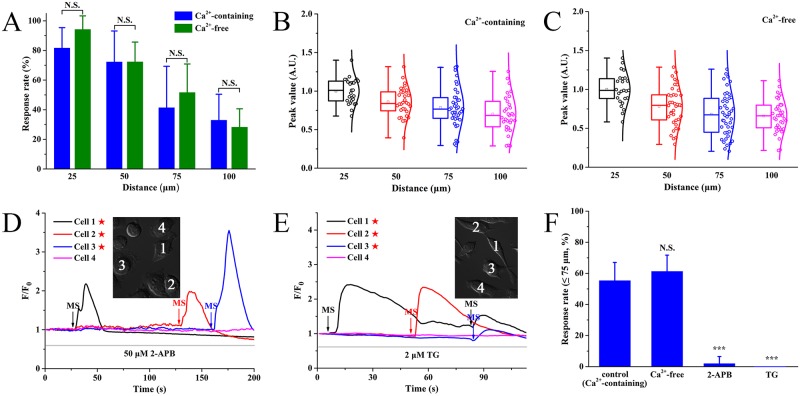
Mechanical stimulation-induced ICWs were dependent on release of IP_3_-sensitive calcium store. MS indicates the mechanical stimulation event. Pentagram indicates the stimulated cell. **(A)** The response rate of ICWs in the presence and absence of extracellular Ca^2+^ (n ≥ 20 cells for each distance from five independent experiments). All values are expressed as mean ± SD. Data are statistically analyzed by the unpaired Student’s t-test. N.S., no significant difference. **(B and C)** The distribution of ICW peak values (n ≥ 25 cells for each distance from six independent experiments) is shown in box plot with and without extracellular Ca^2+^. The mean peak value of 0–25 μm group are taken as 1, and other groups are normalized to 0–25 μm group. **(D)** Inhibition of ICWs by pretreatment with 2-APB (50 μM for 15 min). **(E)** Pretreatment with 2 μM TG for 5 min inhibits ICW propagation. **(F)** Statistical data of the response rate within 75 μm for all experimental groups (n ≥ 90 cells for each condition from at least three independent experiments). Data are statistically analyzed by one-way ANOVA followed by Scheffé post hoc test. ***P < 0.001.

To simulate local injury in the CNS, we mechanically stimulated BV-2 microglia using glass microelectrodes (Fig 1A). Before stimulation treatment, all cells should be in resting states indicated by stable basal [Ca^2+^]_c_ (F/F_0_ = 1 for Fig 1B). In addition, we paid attention to keeping the integrity of plasma membrane after treatment with mechanical stress tested by propidium iodide staining (S1 Fig). Fig 1B displayed the Ca^2+^ traces of the stimulated cell and five representative neighboring cells. Fig 1C and S1 Movie showed a typical process of ICW propagations among BV-2 microglia in response to mechanical stimulus. It can be found that mechanical stimulation triggered a rapid rise of [Ca^2+^]_c_ that originated from the point of the stimulation and then spread throughout the whole cell body (Fig 1C, 1s and 2s). Upon reaching the cell boundaries, the Ca^2+^ transient propagated to the neighboring cells in a wave-like manner, namely, intercellular calcium waves (ICWs). The velocity of these ICWs was 10.43 ± 2.66 μm/s.

### ICWs are mainly due to Ca^2+^ release from intracellular stores

To verify the contribution of external Ca^2+^, we calculated response rate and [Ca^2+^]_c_ peak value by dividing neighboring cells into four groups with different distances, that was, 0–25 μm group, 25–50 μm group, 50–75 μm group and 75–100 μm group. For Ca^2+^-containing condition, the response rate of ICW was 81.7 ± 13.7% (0–25 μm group), 72.4 ± 20.8% (25–50 μm), 41.5 ± 27.9% (50–75 μm) and 33.0 ± 17.4% (75–100 μm) (blue color, Fig 2A). For Ca^2+^-free buffer, it was 94.3 ± 9.0% (0–25 μm), 72.4 ± 13.3% (25–50 μm), 51.7 ± 19.1% (50–75 μm) and 28.3 ± 12.3% (75–100 μm) (green color, Fig 2A). There was no statistically significant difference in the response rate between the Ca^2+^-containing and the Ca^2+^-free groups. For peak value, the mean of 0–25 μm group was taken as 1, and other groups were normalized to 0–25 μm group. It was 1 ± 0.16 (0–25 μm), 0.86 ± 0.19 (25–50 μm), 0.78 ± 0.26 (50–75 μm) and 0.70 ± 0.24 (75–100 μm) in Ca^2+^-containing buffer (Fig 2B). Similarly, the peak value was 1 ± 0.23 (0–25 μm), 0.78 ± 0.28 (25–50 μm), 0.68 ± 0.28 (50–75 μm), and 0.66 ± 0.21 (75–100 μm) in Ca^2+^-free medium (Fig 2C). Together, we believed that ICWs resulted from mechanical stimulation did not rely on external Ca^2+^. To further address the calcium source of ICWs, cells were treated with 2-aminoethyl diphenylborate (2-APB, potent IP_3_ receptor blocker) and thapsigargin (TG, specific inhibitor of sarcoplasmic/endoplasmic reticulum Ca^2+^-ATPase) prior to mechanical stimulus, respectively. Data showed that mechanical stimulus did not induce ICW propagation in the presence of 2-APB (50 μM for 15 min) (Fig 2D) or TG (2 μM for 5 min) (Fig 2E). The statistical response rate of ICWs by 75 μm was 55.4 ± 11.6% (Ca^2+^-containing), 61.3 ± 10.4% (Ca^2+^-free), 2.0 ± 4.5% (2-APB), and 0 ± 0% (TG), respectively (Fig 2F). In brief, these results indicated that Ca^2+^ waves propagation evoked by mechanical stimulus depended on IP_3_-sensitive calcium store release in BV-2 microglia.

### ICW propagation is mediated by ATP-dependent P2Y purinergic signaling

**Fig 3:**
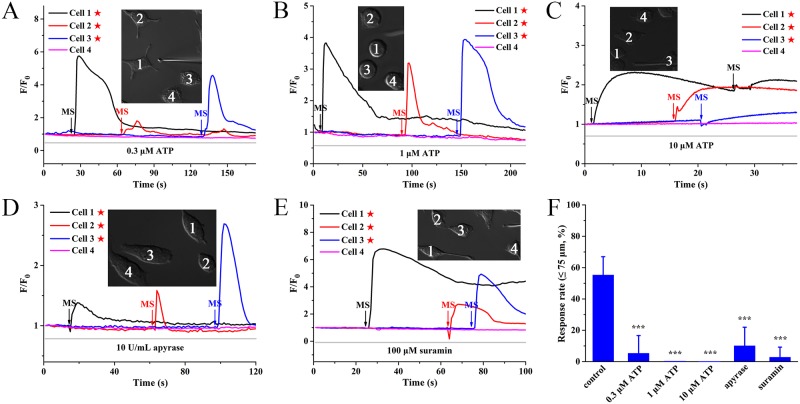
Extracellular nucleotide ATP is associated with ICW propagation via P2Y receptors activation. MS indicates the mechanical stimulation event. Pentagram indicates the stimulated cell. **(A-C)** Application of exogenous ATP (**A**: 0.3 μM; **B**: 1 μM; **C**: 10 μM) blocks mechanical stimulation-induced ICWs in a dose-dependent manner. **(D)** Preincubation with 10 U/mL apyrase for 15 min suppresses calcium waves. **(E)** Pretreatment with 100 μM suramin for 15 min abolishes ICW propagation. **(F)** Statistical data of the response rate within 75 μm for all experimental groups (n ≥ 90 cells for each condition from at least three independent experiments). All values are expressed as mean ± SD. Data are statistically analyzed by one-way ANOVA followed by Scheffé post hoc test. ***P < 0.001.

Our data showed that the 1^st^ treatment with ATP/ADP could not only strongly suppress Ca^2+^ mobilization strength in response to the 2^nd^ challenge, but also significantly delay response time (S2 Fig), indicating that purinoceptors could be desensitized in BV-2 cells with applications of nucleotides. Thus, to investigate whether ICWs were linked to ATP release, we applied different doses of exogenous ATP (0.3, 1 and 10 μM) prior to mechanical stimuli to desensitize corresponding purinoceptors. Results showed that ICWs were largely blocked by 0.3 μM ATP (Fig 3A), and completely suppressed by 1 and 10 μM ATP (Fig 3B and 3C). Besides, preincubation with apyrase (10 U/mL for 30 min), an ATP-hydrolyzing enzyme, evidently blocked the ICWs (Fig 3D), further demonstrating the importance of ATP during ICWs.

Because of the relevance between ATP and P2 purinergic receptors, we determined the involvement of P2 receptors by pretreatment of suramin (a broad-spectrum P2 receptor antagonist). Results showed that ICWs were almost abolished by 100 μM suramin (Fig 3E). As summarized in Fig 3F, the response rate of ICWs (0–75 μm) was 55.4 ± 11.6% for control, 5.4 ± 11.2% for 0.3 μM ATP group, 0 ± 0% for 1 and 10 μM ATP, 10.2 ± 11.8% for apyrase and 2.9 ± 6.4% for suramin, respectively. It was known that P2 receptors could be classified into two families: ionotropic P2X ion channels, which mediate calcium influx, and metabotropic G protein-coupled P2Y receptors that initiate intracellular calcium release via IP_3_-sensitive calcium store [20]. Thus, considering that the ICWs were due to intracellular calcium release (Fig 2), the calcium response was mostly associated with P2Y receptors. These results together demonstrated that the mechanical stimulation-induced ICWs were mediated by extracellular nucleotides ATP via activation of P2Y subtypes in BV-2 microglia.

### The key roles of ADP and P2Y_12/13_ receptors in microglial ICWs

**Fig 4:**
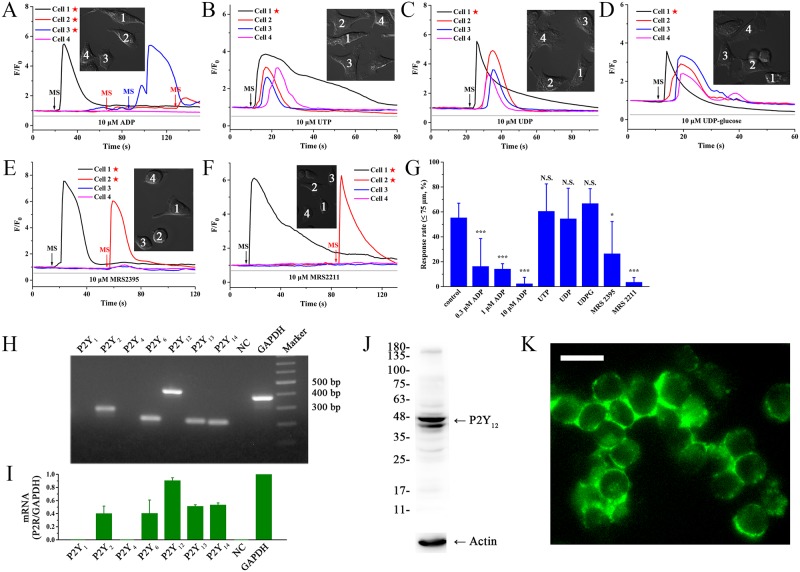
ADP acts as another transmitter associated with activation of P2Y_12/13_ receptors during ICWs. MS indicates the mechanical stimulation event. Pentagram indicates the stimulated cell. **(A)** Application of ADP prior to mechanical stimulation blocks ICWs. **(B-D)** Application of 10 μM UTP **(B)**, 10 μM UDP **(C)** and 10 μM UDP-glucose **(D)** have no inhibitory effects on ICW communication. **(E)** Pretreatment with 10 μM for 15 min MRS2395 partially inhibits ICWs. **(F)** Preincubation with 10 μM MRS2211 for 15 min suppresses ICW propagation. **(G)** Summary inhibitory effects of nucleotides on ICWs within 75 μm (n ≥ 88 cells for each condition from at least three independent experiments). All values are expressed as mean ± SD. *P < 0.05 and ***P < 0.001, one-way ANOVA, Scheffé post hoc test. N.S., no significant difference. **(H)** Gene expression of P2Y receptors in BV-2 microglia at mRNA level detected by RT-PCR. The ten lanes in the gel are as follows: P2Y_1,2,4,6,12,13,14_ (experimental group with primers directed towards the P2YR mRNA); nuclease-free water (negative control); GAPDH (positive control) and marker (with a list of standardized DNA sequences from 100 bp to 800 bp). **(I)** Quantitative statistical results of RT-PCR normalized to GAPDH (n = 3). All values are expressed as mean ± SD. **(J)** Western-blot assay indicates expression of P2Y_12_ receptor in BV-2 microglia. **(K)** Immunolocalization of P2Y_12_ receptor on the membrane of BV-2 cells. Scale bar = 30 μm.

We further tested the effects of other four naturally occurring nucleotides (ADP, UTP, UDP and UDP-glucose) on mechanical stimulation-evoked ICWs. Data showed that ADP could abolish ICW propagation in a dose-dependent manner (Fig 4A and 4G). In contrast, UTP (Fig 4B), UDP (Fig 4C) and UDP-glucose (Fig 4D) had no effect on these ICWs. These results suggested that ADP was also a significant signal molecule during mechanical stimulation-induced ICWs. To investigate the roles of various purinoceptors in ICW propagations, we subsequently examined the expression of different subtypes of P2Y receptors in BV-2 microglia using RT-PCR. Data showed high transcripts of P2Y_12_ receptors, as well as significant expression P2Y_2,6,13,14_ receptors (Fig 4H). The detailed expression rate of mRNA was 40.3 ± 11.3% for P2Y_2_, 40.6 ± 20.2% for P2Y_6_, 90.7 ± 4.0% for P2Y_12_, 51.4 ± 2.1% for P2Y_13_, and 53.6 ± 2.9% for P2Y_14_ (Fig 4I; as the positive control, the expression of GAPDH was taken as 100%). Excluding P2Y_2,4_ (activated by UTP), P2Y_6_ (UDP) and P2Y_14_ receptors (UDP-glucose), therefore, the P2Y_12/13_ receptors, which can be activated and desensitized by ATP/ADP [21, 22], should contribute to ICWs. In order to verify the key roles of P2Y_12/13_ receptors, we performed inhibition experiments utilizing their specific antagonists, MRS2395 (for P2Y_12_R) and MRS2211 (for P2Y_13_R). Results showed that application of 10 μM MRS2395 (15 min) could partially suppress the ICWs (Fig 4E). Meanwhile, 10 μM MRS2211 (15 min) significantly blocked these processes (Fig 4F). As summarized in Fig 4G, the response rate of ICWs (0–75 μm) was 55.4 ± 11.6% for control, 16.4 ± 22.2% for 0.3 μM ADP, 14.2 ± 4.2% for 1 μM ADP, 2.5 ± 5.0% for 10 μM ADP, 60.6 ± 21.9% for 10 μM UTP, 54.6 ± 24.4% for 10 μM UDP, 66.8 ± 11.8% for 10 μM UDP-glucose, 26.5 ± 25.8% for 10 μM MRS2395 and 3.6 ± 3.7% for 10 μM MRS2211, respectively. In addition, we typically confirmed the presence of P2Y_12_ receptor in BV-2 microglia using western-blot assay (Fig 4J) and immunofluorescence (Fig 4K).

### Quantitative estimation of extracellular ATP/ADP concentrations sensed by neighboring BV-2 microglia during Ca^2+^ wave propagation

**Fig 5:**
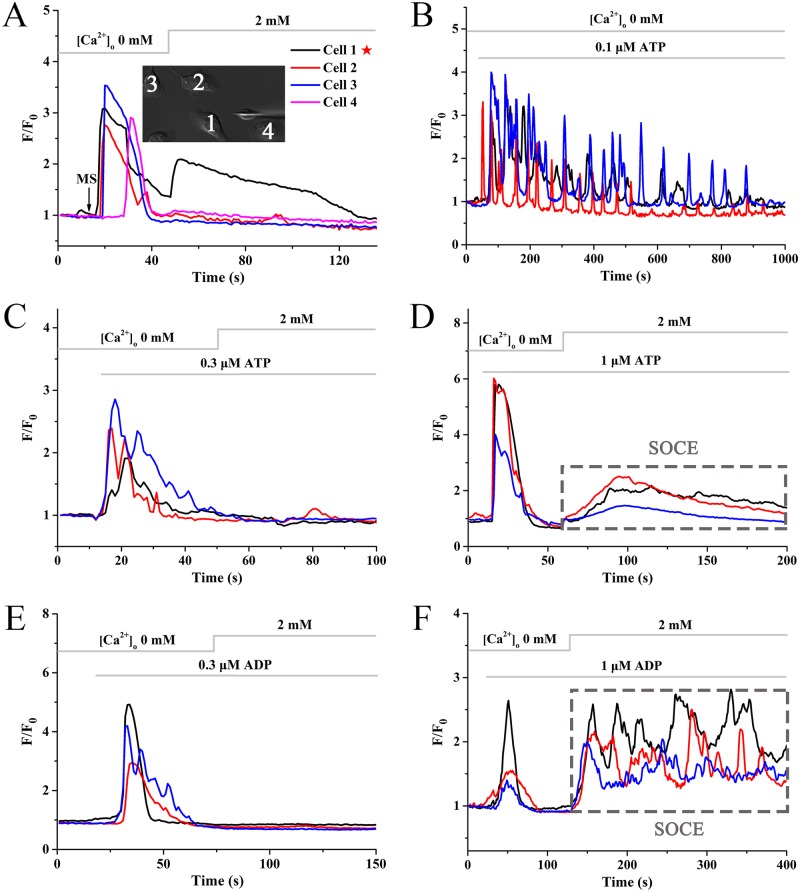
Concentrations of ATP and ADP sensed by P2 receptors during ICWs. MS indicates the mechanical stimulation event. Pentagram indicates the stimulated cell. **(A)** Mechanical stimulus is used to evoke ICWs in the absence of extracellular Ca^2+^, followed by re-addition of 2 mM external Ca^2+^. **(B)** Application of 0.1 μM ATP without extracellular Ca^2+^. **(C and D)** Application of ATP (0.3 and 1μM) in the absence of extracellular Ca^2+^ followed by replenishment of 2 mM external Ca^2+^. **(E and F)** Addition of ADP (0.3 and 1μM) in Ca^2+^-free buffer followed by replenishment of 2 mM external Ca^2+^. Dashed rectangle indicates SOCE.

As a critical mechanism of [Ca^2+^]_c_ regulation, store operated calcium entry (SOCE) can be triggered by a depletion of intracellular calcium stores. Mechanical stimulus-induced ICWs were found to depend on IP_3_ receptor-sensitive stores release in BV-2 microglia (Fig 2). However, readdition of 2 mM external calcium did not lead to a second rising of [Ca^2+^]_c_in non-mechanically stimulated BV-2 microglia (cell 2–4 in Fig 5A), indicating the independence of SOCE. Thus, we used different concentration of exogenous ATP and ADP to mimic the pattern of calcium mobilization during ICWs. Data showed that application of 0.1 μM ATP induced a sustained and regular calcium oscillation in Ca^2+^-free HBSS (Fig 5B). 0.1 μM ADP only resulted in weak Ca^2+^ responses (data not shown). Interestingly, similar to that of mechanical stimulation, application of 0.3 μM ATP (Fig 5C) and 0.3 μM ADP (Fig 5E) induced Ca^2+^ mobilization without SOCE. In contrast, 1 μM ATP (Fig 5D) and 1 μM ADP (Fig 5F) could evoke evident SOCE. Taken together, we suggested that the concentration of ATP and ADP sensed by neighboring BV-2 microglia was about 0.3 μM during mechanical stimulation-induced ICWs.

## Discussion

ICWs appear to be a widespread phenomenon by which various cell types communicate with each other to coordinate and synchronize their activities. For instance, ICWs prefigure and guide the migration of microglia in response to neuronal damage [15], as well as regulate the functions of glial cells in inflammation and immunity [23]. In the present study, we employed mechanical stimulation to an individual BV-2 microglia, and observed remarkable ICWs among adjacent cells. Subsequently, we determined the involvement of several naturally occurring nucleotides in these ICWs. Data showed that ATP and ADP, rather than UTP, UDP and UDP-glucose, were the predominant signal messengers for ICW communication. Furthermore, we indicated that calcium waves were mostly mediated by P2Y_12/13_ receptors.

As significant paracrine messengers in the CNS, nucleotides were known to be released in response to biochemical or physical stimuli, such as oxygen radicals, mechanical forces and virus infection [24, 25]. Nucleotides could be released from the stimulated cells through exocytosis pathway or conductive mechanisms (including pannexin channels, P2X_7_ receptors etc.) [26–28]. The released nucleotides gradually diffused to adjacent cells, which subsequently evoked ICW propagation by activating membrane-bound P2 receptors [29]. In this study, we found that mechanical stimulation triggered remarkable ICWs among BV-2 microglia (Fig 1). Subsequently, we investigated the effects of five naturally occurring nucleotides (ATP, ADP, UTP, UDP and UDP-glucose) on the ICWs. It has been well established that prior challenge with agonists could result in desensitization of P2 receptors, especially the G protein-coupled P2Y receptors to a second challenge with the same agonists [30, 31]. Our results showed that desensitization with ATP (Fig 3A to 3C) and ADP (Fig 4A) significantly blocked mechanical stimulation-induced Ca^2+^ waves. In contrast, application of UTP (Fig 4B), UDP (Fig 4C) and UDP-glucose (Fig 4E) did not abolish ICW propagation. Therefore, these results indicated that ATP and ADP were predominant transmitters during mechanical stimulation-induced ICW communication in BV-2 microglia.

**Fig 6:**
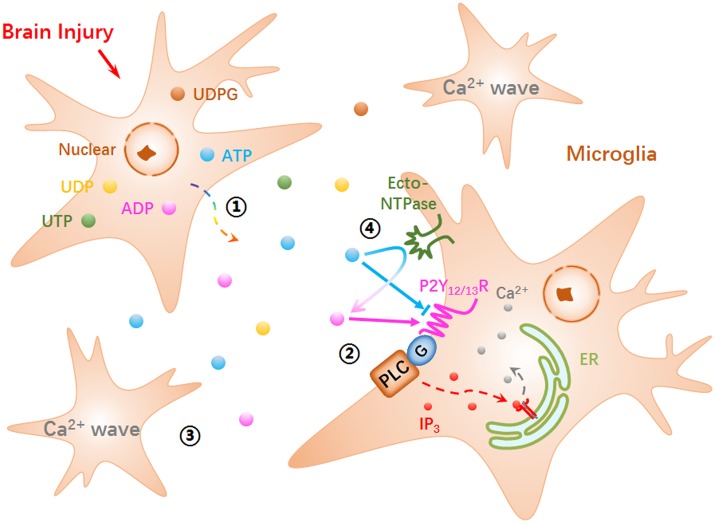
Mechanism underlying mechanical stimulation-elicited ICWs among BV-2 microglia. **①** A range of nucleotides, including ATP, ADP and UDP etc., are released into extracellular space in response to mechanical stimulus. **②** P2Y_12/13_ receptors localized on the membrane of neighboring cells can sense ATP/ADP released from the stimulated cell, then activate PLC/IP_3_/Ca^2+^ signaling. **③** Upon sensing and responding to released ATP/ADP, Ca^2+^ mobilization sequentially occur in neighboring cells, thus perform as intercellular calcium waves. **④** ATP cannot activate P2Y_12/13_ receptors directly. Ecto-NTPase located on the plasma membrane could catalyze hydrolysis of ATP and generates ADP that predominantly activates P2Y_12/13_ receptors.

Furthermore, according to our results, the ICWs were independent on extracellular Ca^2+^ (Fig 2A to 2C) and relied on IP_3_-sensitive calcium store release (Fig 2D and 2E). Thus, it should be attributed to metabotropic G protein-coupled P2Y receptors, rather than ionotropic P2X channels [20]. Among P2Y subtypes expressed in BV-2 microglia (P2Y_2, 6, 12, 13, 14_; Fig 4H and 4I), UTP could activate P2Y_2_ receptors, UDP activated P2Y_6_ and UDP-glucose targeted for P2Y_14_ [21, 22]. In view of the negative effects for these three nucleotides in ICWs (Fig 4B to 4D), P2Y_2,6,14_ could be excluded from the candidate receptors mediating ICWs. On the other hand, the predominantly expressed P2Y_12/13_ receptors, could be activated by both ATP and ADP [21, 22]. Our data showed that desensitizing by ATP/ADP and application of MRS2395/MRS2211 could all eliminate ICWs. Therefore, it determined the pivotal roles of P2Y_12/13_ receptors in ICW communication between BV-2 cells. In addition, although ATP cannot activate P2Y_12/13_ receptors directly [32, 33], membrane-localized ecto-NTPase usually catalyzes hydrolysis of ATP and generates ADP that predominantly activates P2Y_12/13_ receptors [32, 34–36]. Thus, inhibition of ecto-NTPase with ARL 67156 suppressed the ICW propagation in BV-2 microglia (S3 Fig). We summarized an ATP/ADP-P2Y_12/13_R-PLC-IP_3_-Ca^2+^ signaling pathway for mechanical stimulation-induced ICWs by a diagram (Fig 6).

This mechanism we proposed was differ from previous studies, such as P2Y_1,2_ receptors mediated ICWs in rat astrocytes [37], P2Y_11_ receptors contributed to ICWs in rat cardiac myofibroblasts [38] and N-methyl-D-aspartic acid (NMDA) receptor was involved in zebrafish microglial ICWs [15]. Importantly, in addition to participate in ICWs, extracellular nucleotides were associated with a wide array of physiological effects of microglia through activation of specific P2 receptors [39]. For instance, P2X_7_ receptors are relevant to membrane pore formation, microvesicle shedding and cell apoptosis [40, 41]. P2X_4_ as well as P2Y_12_ receptors mediated microglial chemotaxis toward injured sites [42–44], and P2Y_6_ receptor was critical player in promoting microglial phagocytosis resulted from Ca^2+^ mobilization [34].

Metabolic G protein-coupled P2Y receptors are divided into two classes: G_q_ protein-coupled receptors, mainly P2Y_1-11_ receptors, and G_i_-coupled P2Y_12-14_ receptors. Activation of G_q_-protein could stimulate phospholipase C β (PLCβ), then cleave PIP_2_ into two types of second messengers IP_3_ and DAG, and triggered Ca^2+^ mobilization [45]. In contrast, activation of G_i_-coupled P2Y_12/13_ receptors inhibited the adenylate cyclase (AC)/cAMP-dependent pathway [44]. Meanwhile, G_i_-coupled P2Y_12/13_ receptors resulted in activation of the phosphatidylinositol 3’-kinase (PI3K) pathway, which was known to be a crucial enzyme in the regulation of chemotaxis [46]. Nevertheless, some studies reported that activation of P2Y_12/13_ receptor could also mediate PLC/IP_3_/Ca^2+^ pathway [47, 48]. Our study indicated the correlation between PLC/IP_3_/Ca^2+^ signaling and P2Y_12/13_ receptors, which were consistent with de Simone’s [48] and Zeng’s work [49].

Subsequently, we estimated the concentrations of paracrine ATP and ADP sensed by BV-2 microglia during the ICW. The traditional method detecting concentration of ATP was luciferin-luciferase assay. However, this method was mostly used for the measurements of the overall level of ATP concentration and not applicable to ADP. In this work, we found that mechanical stimulation-evoked ICWs was due to calcium release rather than SOCE (Fig 5A). Therefore, we observed whether the [Ca^2+^]_c_ pattern in response to exogenous ATP and ADP at different concentration were in agreement with mechanical stimulation-induced ICW. Data showed that different concentration of nucleotides could induce different patterns of Ca^2+^ transient, which was similar to that of Visentin’s work [50]. Only application of 0.3 μM ATP or ADP exhibited a similar characteristic behavior to that of mechanical stimulation-evoked ICWs (Fig 5C and 5E), indicating that the concentration of ATP and ADP sensed by purinergic receptors of BV-2 microglia was about 0.3 μM. These findings might be significant in the field of drug analysis and development and could provide the basis for pharmacological experiments.

One of the main limitation on this study was the application of BV-2 microglia. Although BV-2 has been known as an immortalized mouse microglial cell line that exhibits the morphological and functional characteristics of primary microglia [19, 51], there is no denying that it cannot fully represent the real physiological features of primary microglia *in situ* or *in vivo* [52]. Therefore, we plan to study and understand the procedure of primary microglia isolation. Much work will be performed on primary microglia that makes our research more interesting and significant.

In summary, our study demonstrated that ATP and ADP were predominant signal transmitters in mechanical stimulation-induced ICWs through P2Y_12/13_ receptors activation in BV-2 microglia. The concentration of ATP/ADP sensed by BV-2 microglia was about 0.3 μM. Our research may bring new insights in the mechanism of cell-to-cell communication of microglia to investigation of its immune defense in the CNS.

## Supporting information

S1 Movie Intercellular calcium wave triggered by mechanical stimulus in BV-2 microglia. (AVI)

S1 Fig BV-2 microglia maintain or lose plasma membrane integrity after different mechanical stimulations. (DOCX)

S2 Fig Desensitization of purinoceptors by application of ATP and ADP. (DOCX)

S3 Fig Ectonucleotidase inhibitor ARL 67156 significantly blocks the ICW propagations in BV-2 microglia. (DOCX)

S1 Table Primers used to detect P2Y receptor genes in BV-2 microglia in RT-PCR. (DOCX)
