# Supplementary material for: Nucleotide transmitters ATP and ADP mediate intercellular calcium wave communication via P2Y12/13 receptors among BV-2 microglia
Source: PLoS One. 2017 Aug 11;12(8):e0183114. doi: 10.1371/journal.pone.0183114 (PMC5553643; doi:10.1371/journal.pone.0183114)
Supplement: S1 Fig — (DOCX) [file pone.0183114.s002.docx]

**S1 Fig. BV-2 microglia maintain or lose plasma membrane integrity after different mechanical stimulations. (A)** Representative image of BV-2 microglia before mechanical stimulation visualized by DIC microscope. The white arrowheads indicate the stimulated cells. **(B)** Suitable mechanical stress just makes a certain change in profile of BV-2 microglia (white arrowheads). However, unsuitable mechanical stress not only causes the cell to undergo severe deformation, but also results in membrane blebbing (yellow arrowhead). **(C)** BV-2 cells stained with propidium iodide (PI, red). Only one cell indicated by yellow arrowhead uptakes PI dyes, indicating that unsuitable mechanical stress can directly lead to cell death. **(D)** Merge of bright field and fluorescence images. Scale bar = 30 μM.

**
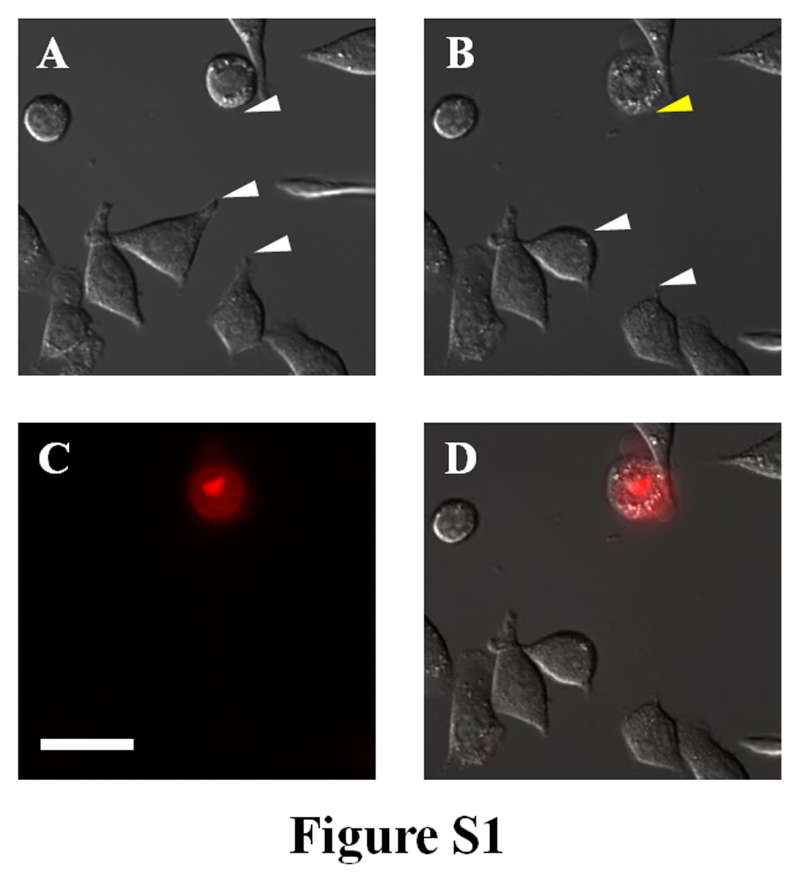
**
