# Supplementary material for: Nucleotide transmitters ATP and ADP mediate intercellular calcium wave communication via P2Y12/13 receptors among BV-2 microglia
Source: PLoS One. 2017 Aug 11;12(8):e0183114. doi: 10.1371/journal.pone.0183114 (PMC5553643; doi:10.1371/journal.pone.0183114)

**S2 Fig. Desensitization of purinoceptors by application of ATP and ADP**. (**A**) 1 μM ATP evidently inhibits Ca^2+^ mobilization strength in response to a second challenge with 1 μM ATP. (**B**) The response rate is 98.8 ± 1.9 % (1^st^ ATP treatment) and 26.1 ± 3.0 % (2^nd^ ATP treatment), respectively (n=100 cells from three independent experiments). All values are expressed as mean ± SD. Data are statistically analyzed by the unpaired Student’s t-test. ****P* < 0.001. (**C**) Box-plot of the delay time for the 1^st^ ATP (3.9 ± 1.2 s, n=98) and the 2^nd^ ATP (25.0 ± 9.0 s, n=25). (**D**) 1 μM ADP strongly abolishes Ca^2+^ response intensity to a second stimulation with 1 μM ADP. (**E**) The response rate is 100 ± 0 % (1^st^ ADP treatment) and 3.7 ± 2.6 % (2^nd^ ADP treatment), respectively (n=120 cells from three independent experiments). All values are expressed as mean ± SD. Data are statistically analyzed by the unpaired Student’s t-test. ****P* < 0.001. (**F**) Box-plot of the delay time for the 1^st^ ADP (1.9 ± 0.7 s, n=120) and the 2^nd^ ADP (16.0 ± 5.4 s, n=4). 1 μM ATP (**G**) and 1 μM ADP (**H**) completely block Ca^2+^ increases induced by 0.3 μM ATP and 0.3 μM ADP, respectively.


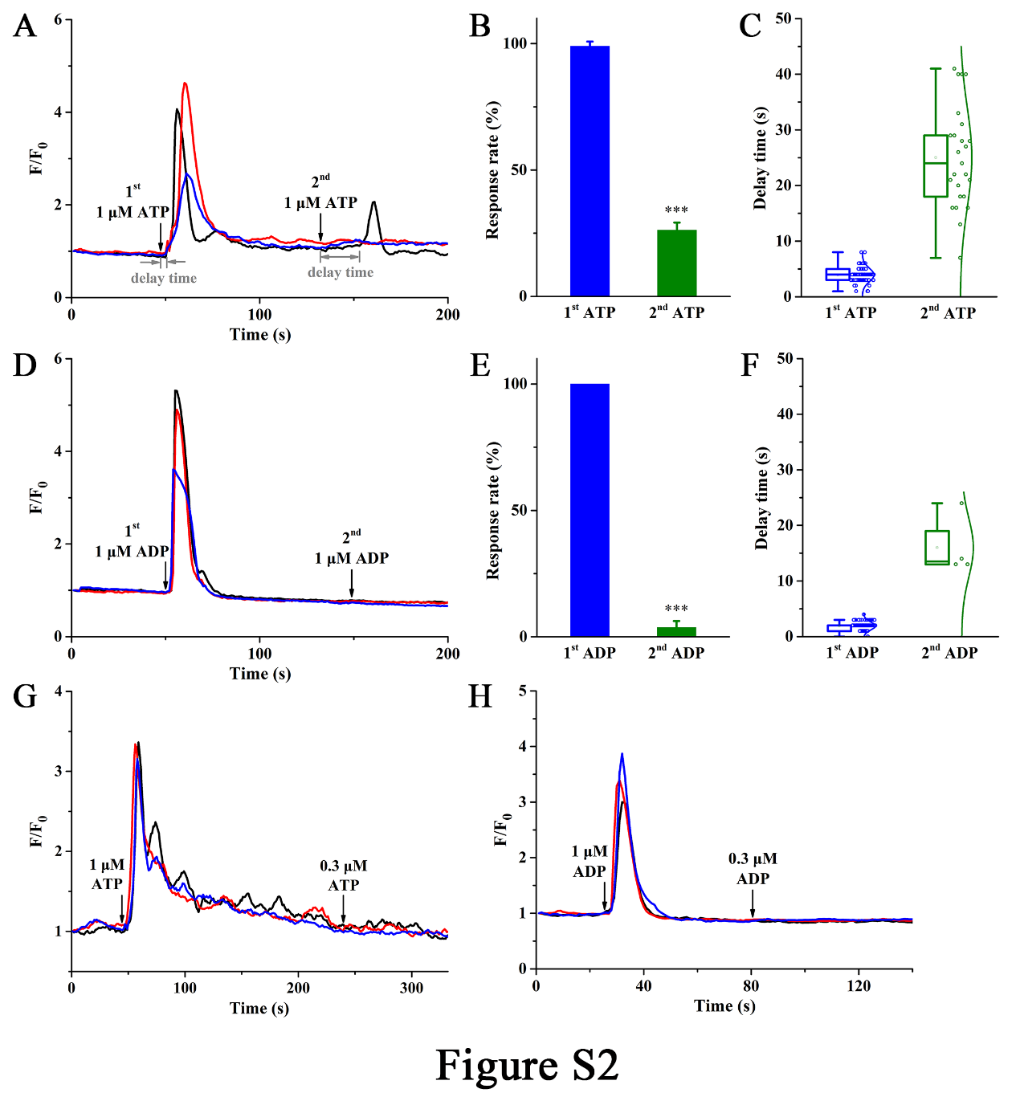

Supplement: S2 Fig — (DOCX) [file pone.0183114.s003.docx]
