# Supplementary material for: Nucleotide transmitters ATP and ADP mediate intercellular calcium wave communication via P2Y12/13 receptors among BV-2 microglia
Source: PLoS One. 2017 Aug 11;12(8):e0183114. doi: 10.1371/journal.pone.0183114 (PMC5553643; doi:10.1371/journal.pone.0183114)
Supplement: S3 Fig — (DOCX) [file pone.0183114.s004.docx]

**S3 Fig. Ectonucleotidase inhibitor ARL 67156 Significantly blocks the ICW propagations** **in BV-2 microglia.** MS indicates the mechanical stimulation event. Pentagram indicates the stimulated cell. (**A**) Application of ARL 67156 (100 μM for 10 min) blocks ICW propagations. (**B**) The response rate of ICWs within 75 μm is 55.4 ± 11.6 % for control and 3.3 ± 10.9 % for ARL 67156 (n ≥ 57 cells from three independent experiments). All values are expressed as mean ± SD. Data are statistically analyzed by the unpaired Student’s t-test. ****P* < 0.001.

**
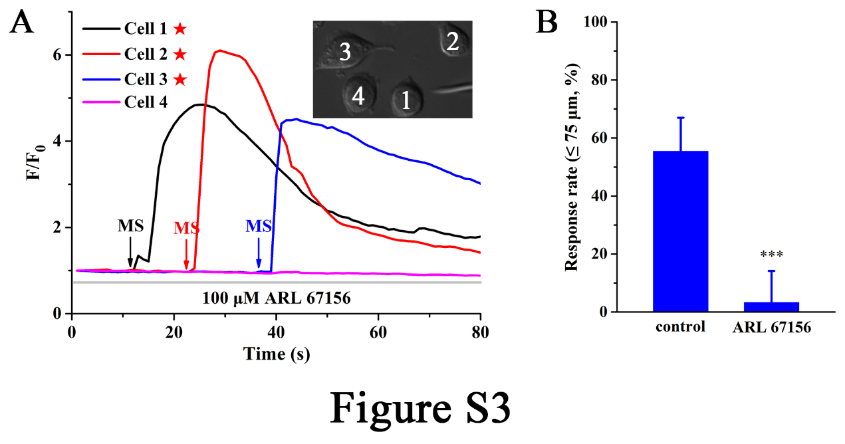
**
