# Supplementary material for: Nucleotide transmitters ATP and ADP mediate intercellular calcium wave communication via P2Y12/13 receptors among BV-2 microglia
Source: PLoS One. 2017 Aug 11;12(8):e0183114. doi: 10.1371/journal.pone.0183114 (PMC5553643; doi:10.1371/journal.pone.0183114)
Supplement: S1 Table — (DOCX) [file pone.0183114.s005.docx]

**S1 Table. Primers used to detect P2Y receptor genes in BV-2 microglia in RT-PCR.**

| **Target** | **Pubmed acc. No.** |  | **Primer sequence** | **Predicted length (bp)** |
| --- | --- | --- | --- | --- |
| P2Y_1_ | NM_008772.5 | Forward  Reverse | TGGCTATCTGGATGTTCG  TCGCAGGTAATCATTGGAC | 425 |
| P2Y_2_ | NM_008773.4 | Forward  Reverse | ACCCTCAACGCCATCAAC  AGCCGAATGTCCTTAGTCTCAC | 281 |
| P2Y_4_ | NM_020621.4 | Forward  Reverse | ACTCTGGTCTGCTATGGA  ATCCACCAAGACAGCATC | 423 |
| P2Y_6_ | NM_183168.2 | Forward  Reverse | ACCACCTGCGTCTACCGT  GTCCCCTCTGGCGTAGTTAT | 225 |
| P2Y_12_ | NM_027571.3 | Forward  Reverse | GACCGCTACCTGAAGACCAC  CAGGGTGTAGGGAATCCGTG | 423 |
| P2Y_13_ | NM_028808.3 | Forward  Reverse | TCGCTTTCGACAGGTTCCTC  TGTGACTGACCACCTGATGC | 222 |
| P2Y_14_ | NM_001008497.2 | Forward  Reverse | CTGCTTTCTGTGCTCGTGTG  CGTGATGGCCGTGTAGAAGA | 213 |
| GAPDH | NM_001289726.1 | Forward  Reverse | GCATTGTGGAAGGGCTCA  AAGGTGGAAGAGTGGGAGTT | 379 |
